# Poles Apart? The Extent of Similarity Between Online Extremist and Non-extremist Message Content

**DOI:** 10.3389/fpsyg.2021.776985

**Published:** 2021-11-19

**Authors:** Sheryl Prentice, Paul J. Taylor

**Affiliations:** Department of Psychology, Lancaster University, Lancaster, United Kingdom

**Keywords:** extremism, counter-extremism, mainstream, (dis)similarity, positioning, resistance

## Abstract

Within studies of extremism, extremist and non-extremist messages are generally treated as two sets of competing constructed narratives. However, some research has argued that these message forms are not dichotomous and that non-extremist narratives demonstrate overlap with extremist master narratives. The aim of this paper is to test this hypothesis empirically by comparing 250 extremist, 250 mainstream and 250 counter-extremist messages. The paper finds considerable overlap between extremist and non-extremist material. However, an analysis of underlying content suggests that this overlap may not be so much due to the extensive adoption of an extremist master narrative by non-extremist authors, but rather a question of resistance and positioning, specifically, who are authors resisting and why? The findings have implications for counter-extremism policy.

## Introduction

Master narratives are “dominant cultural storylines which form the context of (people’s) lives” and are the means by which we understand our own stories and those of others, “identifying what is assumed to be a normative experience” (Andrews, 2004, p. 1). With reference to the work of Halverson et al. (2011), Al Raffie (2012) describes how a type of extremist master narrative (namely, Salafi Jihadist master narratives) have gradually attempted to reshape the normative experience of Muslims by basing themselves on well entrenched Muslim cultural master narratives, which are built on religious texts and Muslim history. Salafi Jihadist master narratives are said to be characterized by the creation of “both real and perceived hostilities between Muslims and non-Muslims; cementing a perception of a “War on Islam,” which ultimately seeks to divide Muslims and non-Muslims via a religious filter (Al Raffie, 2012, p. 19).

Drawing on the work of Huband (2010), Al Raffie (2012, p. 15) explains that this goal is achieved via reference to a politically and sociologically dominating situation, linking religious sources to the sociological situation, and constructing identity as the result of these two factors. According to Al Raffie (2012, p. 25), this attempt to reshape Muslims’ normative experience has been adopted by the mainstream and receives support from a range of organizations, states and actors, going on to argue that “the only difference between them and Salafi Jihadist narratives is that they are more strategic in communicating their desired end effects and seemingly reject violent tactics.” This paper seeks to empirically test the hypothesis that Salafi Jihadist narratives, and those of other groups and individuals advocating a similar message, are present in mainstream narratives, and to what extent, by comparing sets of extremist and non-extremist messages.

The paper begins by reviewing literature on the similarities and differences between extremist and non-extremist messages, before moving to a description of the collection and comparison of four inter-related message forms: Salafi Jihadist (and related) messages, mainstream news articles from Arab based media outlets, religious authored counter-extremist messages and British Official authored counter-extremist messages (as a control). The extent and nature of conceptual overlaps between the forms of material is discussed before examining how authors position themselves relative to shared concepts. The concluding discussion of the paper explores how work on narratives of resistance can best explain the similarities observed between extremist and non-extremist message forms.

This work is a combined result of research (see Prentice, 2013) undertaken as part of the *Time, Response and Audience Construed Evaluation of (Counter-)Extremist Messages (TRACE)* project (funded by HMG) and the *Building Resilience Against Violent Extremism and Polarisation (BRaVE)* project (funded by the European Commission’s Horizon 2020 research and innovation programme, Grant no. 822189).

## Background

Typically, research on extremist material, or research comparing extremist and non-extremist material, seeks to understand what is unique about extremist forms of communication. Research treating extremist and non-extremist language as opposing entities is grounded in the theoretical assumption that extremists possess unusual ways of thinking (Pearlstein, 1991; Johnson and Feldmann, 1992; Merari, 1999; Merari et al., 2009), or a differing psycho-logic (see Post, 1990). If one holds to the assertion that language is one of the key ways in which the thoughts and beliefs of individuals are reflected (Billig, 1997; Pennebaker, 2002; van Dijk, 2006), it follows that extremist language would be markedly different from non-extremist language, since it presumably reflects an alternative way of thinking about the world.

Applying this to the language used by proscribed terrorist groups in the United Kingdom (specifically, those advocating a violent interpretation of Jihad), studies have found differences between the communications of such groups and those of control groups. Prentice et al. (2012a), for example, identified content differences between a corpus of religious extremist statements and a corpus of general English usage. They found that extremist authors center their rhetoric on the themes of morality, social proof, inspiration and appeals to religion, and that they tend to refer to the world via contrasting concepts, suggesting a polarized way of thinking when compared to a general population usage.

Similarly, Payne (2009) has identified differences between the narratives of Al-Qaeda authors and opposing Western government authors. He found that Al-Qaeda’s narrative is characterized by the concepts of Islamic utopia, an “us-versus-them” dichotomy, jihad as a just response, legitimizing terrorism and glorifying martyrdom. By contrast, government narratives were characterized by the concepts of undermining Al-Qaeda and building resilience and community cohesion through a sense of “Britishness.” Payne’s (2009) findings demonstrate a second, more overt reason why the content of extremist and non-extremist messages should differ: The authors of these messages may deliberately seek to distance their rhetoric from one another for strategic purposes.

Some researchers have argued that in order to counter the risk posed by extremist rhetoric, non-extremist message content should directly oppose the arguments made in extremist messages by delegitimizing political violence and the actors who pursue it, thereby creating their own form of counter-persuasion (Halafoff and Wright-Neville, 2009; Chowdhury and Krebs, 2010; Gregg, 2010). Likewise, Awan (2007) has found that extremist sources present a differing perspective to mainstream non-extremist sources in an effort to challenge the latter’s hegemony. Therefore, whether unintentionally reflecting differing thought processes, or intentionally distancing themselves from one another’s arguments, extremist and non-extremist message content is, under this popular conceptualization, expected to differ.

There are, however, reasons to believe that the narratives of extremist and non-extremist messages are not as directly opposed as the aforementioned literature implies. Mainstream media can be observed to take on Gutmann’s (2007) qualities of extremist literature, in that press articles have been found to demean perceived out-groups and narrow understanding of particular individuals (such as asylum-seekers or Muslims) or social issues (including immigration and practicing Islam) (see Richardson, 2004; Baker, 2010; for examples). Press reports have further been found to legitimize and remediate extremist actors and their arguments (Al-Marashi, 2007; Azam, 2008; Hoskins and O’Loughlin, 2009).

Mainstream political language has also been observed to adopt a number of similar rhetorical strategies to extremist authors. Schafer (2002); Leudar et al. (2004), and Jones and Smith (2010) for example, have all identified unifying terms of reference (i.e., “we,” “us,” etc.) to create an in-group in the language of both Western secular and extremist authors as they vie to achieve success in winning over public opinion. These in-group and out-group discourse features have been further noted in the language of Western politicians (Lazar and Lazar, 2004; Becker, 2007; Richardson and Wodak, 2009; Verkuyten, 2013). Non-extremist political language holds additional aspects in common with extremist language in its moral and social justificatory arguments for warfare, which have been observed in both political (Lazar and Lazar, 2007) and extremist statements (Duffy, 2003).

There are a few reasons why extremist and non-extremist rhetoric may overlap. Numerous studies have demonstrated that sharing various identity-related factors, such as race, ethnicity and religion can result in individuals converging their language features (Labov, 1972; Cheshire, 1997; Milroy and Milroy, 1997; Joseph, 2004). Such sociolinguistic research links with social identity theory’s assertion that people identify themselves as belonging to particular groups, using group norms to enforce membership of groups, and boundaries with other groups (Tajfel, 1978, 1982; Tajfel and Turner, 1979). Language is one of the ways in which these social identities are achieved and maintained (Billig, 1997).

Indeed, Prentice et al. (2012b) have found ideological content links between religious extremist and religious counter-extremist messages, such as descriptions of the legitimacy of violence in circumstances defined by their mutual faith (see also Khān, 2002; Mascini, 2006). Bilali (2014) has also observed an association between national identification and conflict construal across the narratives of Turkish and Kurdish ethnic groups. A linked explanation for the rhetorical overlaps observed between extremist and non-extremist messages emerges from Zaal et al. (2011), who have found that individuals “with a strong moral conviction about the fair treatment of their group are willing to support both hostile and benevolent forms of collective action.”

Such theories may explain the adoption of Salafi Jihadist (and related) master narratives by mainstream voices observed by Al Raffie (2012). The ultimate aim of this paper will be to quantify the extent of any relationship between extremist and non-extremist narratives and to qualify whether any observed relationship is due to the adoption of extremist master narratives by mainstream authors on the grounds of religious in-group identification.

## Materials and Methods

This section details the collection of four corpora of religious extremist, mainstream, counter-extremist and control messages, and the procedure used in their analysis.

### Corpus Collection

Analyses were conducted on four corpora: A 425,516 word extremist message corpus, containing 250 texts written by members of religious extremist groups or unaffiliated extremist individuals (*M* = 1814.0 words, *SD* = 2327.1); a 107,018 word mainstream message corpus containing 250 news articles drawn from four popular middle-eastern news outlets (*M* = 446.0; *SD* = 254.1), a 119,678 word religious counter message corpus, containing 200 anti-violent messages from Muslim clerics and discussion boards (*M* = 598.4, *SD* = 731.6), and a 89,254 word British Official authored counter message corpus, containing 50 statements authored by British politicians (*M* = 1785.1, *SD* = 1763.7).

The religious and British Official counter messages were originally collected (by MacInnes, 2014) as one corpus of 250 messages. However, as this study aims to determine whether there is narrative overlap between extremist and non-extremist authors who identify with the same religion, the messages are considered separately here. British Official messages are included as a control group.

All data sets feature English language messages because of their use by extremist groups to appeal to the widest possible audience (Memri Organization, 2007). All messages are drawn from online sources, due to an increasing tendency for this community to utilize online sources for information gathering and distribution (Brouwer, 2004; Hirji, 2006). Collection of messages for the extremist data set began with targeting the websites of known extremist organizations and individuals in, for example, the HM Government (2012) list of proscribed terrorist groups and organizations.

This was followed by an investigation of links from such websites to other sites containing extremist material. Specifically, of the 250 messages, 160 were drawn from the websites of 15 different extremist groups and organizations (such as Al-Qaeda), and the remaining 90 from the websites of 67 unaffiliated individuals (such as Al-Fallujah forums). To be included, messages had to explicitly advocate the use of violence (this is due to our interpretation of extremist messaging, i.e., the incitement of violence against civilians), thus avoiding the inclusion of messages in which authors only sought to advocate a strict version of their beliefs, where the boundaries between extreme and non-extreme material become increasingly blurred. The messages are dated between 1996 and 2009.

The 200 religious counter messages and 50 British Official authored counter messages originate from MacInnes (2014) and are largely from counter-extremist websites affiliated with counter-extremist individuals within Muslim communities. The messages combine anti-violent responses from religious scholars to guest questions on the use of violence (94 texts) and anti-violent open discussion forum posts on topics of violence (106 texts). The 50 British Official counter messages consist of British officials’ statements, collected from news sites or government websites.

In MacInnes’ (2014) study, authors had to be recognizable public figures whose statements would be regarded as espousing the position of the United Kingdom government. Their inclusion provides an alternative perspective on the issue of counter-extremism, a perspective that is also important to British Muslim identity (Pew Research Centre, 2006). Further, Al Raffie (2012) states that the position in such messages lends legitimacy to an extremist master narrative by way of apology and confirmation of wrong-doing. Therefore, their inclusion offers a means of exploring whether this is the case. Further, British Official Counter messages are included because the paper discusses the hypothesis that extremist and non-extremist, moderate authors who identify with the same religion in this case, will demonstrate similar language use. That being the case, one should not then observe extensive overlap with individuals who do not identify with the same religion (i.e., the British Official authors included). The longer length of the British official messages means that increasing their number would over-represent this secondary perspective in the data.

Finally, a mainstream corpus was created to contribute a perspective that is neither directly pro- or anti- violence. News articles, specifically, current affairs articles, were selected for this purpose, as they have been identified as a common and credible source of information in studies of Muslims’ media consumption more generally (Next Page Foundation, 2007). Data was sourced from Al Jazeera (94 texts), Press TV (63 texts), Al Arabiya (63 texts), and Al Alam (30 texts). These sources were selected as they have been observed to be credible to one or more Muslim communities within the United Kingdom (RICU, 2010). These data were downloaded from the news and current affairs sections of the respective sites. Selection of texts from the four sites was weighted according to site reputation, i.e., the number of other sites linking into the site, making it more likely to be viewed by a wider audience (reputation rankings were drawn from www.alexa.com/siteinfo). Texts were selected at random for inclusion in the corpus in order not to bias text selection. More specifically, the filenames associated with lists of downloaded articles from the news/current affairs section of each website were extracted and an automated randomization algorithm used to select the weighted number of articles from each source. Texts had to be at least 100 words in length. Where a randomly selected article failed to meet this criterion, the same algorithm was used to select an alternative.

Given the subject matter, one might question why counter-messages have been included in an analysis of extremist and mainstream narrative overlap. The reasons for including counter-extreme messages in the analyses are twofold. First, counter-extreme messages are interpreted here as another form of “non-extremist” message, or moderate/mainstream voice. Their inclusion therefore allows for the comparison of extremist narratives with different types of “non-extremist” narrative, both those that are directly non-extreme in nature (counter-extremist) and those that are indirectly non-extreme (mainstream news reporting). Second, if one were to only consider how mainstream media overlap with extremist material, one would ignore its potential to overlap with the antithesis to this content (i.e., counter-extreme material).

### Content Coding

The texts were examined using the semantic analysis software Wmatrix. Wmatrix works by labeling every word or multi-word-unit (MWU) in a text file for its part-of-speech and semantic category. The part-of-speech tagger (named CLAWS) assigns major word class categories (e.g., noun, verb, adjective, and adverb) to each linguistic unit (defined as single words and multi-word-expressions) in a text. The semantic tagger USAS uses a manually created dictionary (Piao et al., 2005) and several word sense disambiguation techniques (Rayson et al., 2004a) to assign the same linguistic units to one or more of its 232 semantic categories. These categories (a full list of which can be found at ucrel.lancs.ac.uk/usas/) are classified into 21 broad domains, or groups of semantically related terms (see ucrel.lancs.ac.uk/usas/ for all domains).

To give an example, in the sentence “The Prime Minister visited Afghanistan,” “The” would be assigned to Grammatical words, “Prime Minister” to Government and People, “visited” to Social actions, states and processes and Moving, coming and going, and “Afghanistan” to Geographical names. The category and domain-based classifications allow the user to conduct both macro (domain) level and micro (category) level analyses of the data using a variety of statistical methods.

Wmatrix’s automated approach was adopted over a manual approach to ensure continuity in the application of codes across the three corpora. Although other automated approaches have proved useful in previous studies involving extremist material (Pennebaker and Chung, 2008; Bermingham et al., 2009), the distinct advantage offered by the Wmatrix package is the granularity of its coding systems, allowing both macro and micro level analyses of the data (see, for example, Rayson, 2008).

### Keyness Comparison Procedure

Once processed by Wmatrix, it was possible to retrieve semantic category lists for each of the four corpora. The lists contained the semantic categories present in each corpus together with their frequency of occurrence. These lists were then submitted to a form of analysis known as keyness comparison, which in this case involves two steps. The first step of keyness comparisons is to identify categories that are over or underused beyond what might be expected by chance. To determine this, the log-likelihood value of each semantic category’s frequency of occurrence across the corpora was calculated.

By calculating the log-likelihood value for each category across the four corpora, it was possible to establish the number of categories being significantly overused or underused in a particular corpus or corpora, relative to the others. These significant categories, therefore, highlight the aspects of content on which the corpora significantly differ from one another. Any log-likelihood value of 15.14 (*p* < 0.0001) is deemed to be statistically significant in the present study. As log-likelihood measures can generally skew one’s data in the direction of differences, alongside this measure, approximate Bayes Factors (BIC) are used to calculate effect size, with BIC values > 10 indicating very strong evidence against the null hypothesis of no difference between the corpora on a given category and BIC values < –10 indicating very strong evidence in favor of the null hypothesis (see Wilson, 2013).

Therefore, in the present study, any category with a log-likelihood value of ≥ 15.14 and a BIC value of ≥ 10 was counted as indicating a difference between corpus sets, while any category with a BIC value of ≤ –10 was counted as indicating no difference between the comparison corpora. As low corpus frequencies (i.e., ≤ 5) have been found to affect the usefulness of the log-likelihood statistic (Rayson et al., 2004b), any categories where a corpus (or corpora) returned a frequency ≤ 5 were removed from the analysis.

While this analysis reveals the areas of difference and similarity between all the corpora, it does not determine the corpus responsible for the differences, which would in turn highlight aspects of content held in common by the remaining corpora. To achieve this, the second step is to calculate the under and over use of each category in each corpus. In this case, if the observed frequency of a category in a particular corpus was less than its expected frequency, this was classed as underuse of the category. By contrast, observed frequencies greater than expected frequencies were recorded as being overused. For further details on this method, see Prentice et al. (2021).

Overused categories for each corpus, corpus pair, or corpus trio were taken to be characteristic of the corpus/corpora in question and summed to give a profile for each corpus comparison. The percentage of categories above the designated threshold assigned to each individual corpus or corpus grouping were then compared to establish which corpus/corpora accounted for the greatest number of shared conceptual categories. Shared categories for these corpora were then listed and examined to gain an overall understanding of the nature of conceptual overlap between particular message types.

### Semantic Concordance Analysis

While the adaption of the keyness comparison method outlined above identifies the extent and nature of shared concepts between the corpora, which can offer initial indications as to whether message types share narratives with one another, one can only confirm this by exploring the context in which concepts occur. Specifically, our analysis looks at how authors of messages position themselves in relation to shared concepts. Who do the authors identify with, who is their audience, and who is the out-group?

After running the corpora through part-of-speech and semantic tagging, various frequency lists are made available to the user via Wmatrix’s interface. This includes a list of words, along with their semantic category and frequency of occurrence in a corpus. These lists were used to source the most frequently occurring word assigned to each shared category. Once located, Wmatrix’s concordance function was used to search for the word and provide a list of examples of the word in its immediate linguistic context. Examples were selected at random and can be found in Tables 1–4.

**TABLE 1:** Examples of shared conceptual categories between Salafi Jihadist/Related messages (Extremist), religious authored counter-extremist messages (Muslim_Counter), Arab Mainstream Media messages (Mainstream), and British Official counter-extremist messages (BrOfficial_Counter).

**Category**	**Corpus**	**Example**
B4	Extremist	“Verily, the sword does not **wipe** off an-Nifaaq (hypocrisy)”
	Muslim_Counter	“This includes struggling against evil inclinations and **purifying** one’s soul”
	Mainstream	“‘These events can no longer be **swept** under the carpet. If followed by strong regional and international action, this report could make a major contribution to ending the impunity that lies behind the cycle of atrocities in the Great Lakes region of Africa,’ he added.”
	BrOfficial_Counter	“But the narrative of grievances has sufficient plausibility that it cannot just be **brushed** aside”
L3	Extremist	“Accordingly, although Muslims have divided themselves into sects, nonetheless, a way out is that we should be united like a huge **tree** which has numerous **branches**, they are not disconnected”
	Muslim_Counter	“Actually, after reading the news, one realizes why the ‘civilized world leaders’ might never succeed in stopping terrorism! For one thing, they do not want to hear about the **root** causes of terrorism”
	Mainstream	“This is the will of the regional nations that after 60 odd years, the **root** of this corrupt microbe and the main reason for insecurity in the region be pulled out”
	BrOfficial_Counter	“I thought then and I think now that defeating this threat—whose **roots** are deep and have been a long time growing—was going to take a generation”
N4	Extremist	“**Then** there was the coordination after Afghanistan, to eliminate the former Iraqi regime”
	Muslim_Counter	“Both sides of the argument should be heard, the situation should be analyzed, and the reason and the intention of the person should be taken into account, and **then** the person can be judged accordingly”
	Mainstream	“Netanyahu, who has said he would push hard to clinch a deal, also wants the U.S. letter to spell out that the proposed moratorium would be the **last**”
	BrOfficial_Counter	“**First**, in this country. The unavoidable priority is to identify the individuals who intend to commit violent acts and prevent them doing damage”
H4	Extremist	“People **live** in perpetual fear and paralyzing terror, awaiting death at any moment from a missile or shell which will destroy their **homes**, kill their sisters and bury their babies alive”
	Muslim_Counter	“Some countries where Muslims **live** have been attacked and **occupied** in the last few years, so I think it’s not wrong for the population to resist the invasion, but this has nothing to do with putting bombs in trains in Madrid and London”
	Mainstream	“Palestinians said that Israeli **settlers** in the **occupied** West Bank burned about 200 of their olive trees on Sunday and also torched surrounding grazing land”
	BrOfficial_Counter	“But we have to work at finding what we have in common and making this a **home** for all of us”
E1	Extremist	“I was in contact with him and I asked him about his **morale**. He told me he was very happy”
	Muslim_Counter	“For centuries, their tolerance and **compassion** have characterized Muslims”
	Mainstream	“The loss of civilian lives at the hands of foreign forces has dramatically increased anti-American **sentiments** in Afghanistan”
	BrOfficial_Counter	“Over the coming months, in the courts, in parliament, in debate and engagement with all parts of our communities, we will work to turn these **sentiments** into reality”
X7	Extremist	“We don’t **want** oppression. We **want** to regain the freedom of our Muslim nation”
	Muslim_Counter	“We are human beings too. We **want** a peaceful life. Afghans want to be educated and have a prosperous life”
	Mainstream	“‘We no longer **want** military coups in this country. We **want** a civilian and a more democratic constitution,’ said Serkan Misirlioglu”
	BrOfficial_Counter	“We **want** to respect all of our communities, including the Muslim community. But we also **want** to deal with the extremists in our ranks, because that is a way of protecting our way of life”

*Bold font corresponds to a word from a shared category.*

**TABLE 2:** Examples of shared conceptual categories between Salafi Jihadist/Related messages (Extremist) and religious authored counter-extremist messages (Muslim_Counter).

**Category**	**Corpus**	**Example**
S9	Extremist	“All this is happening at a time in which nations are attacking **Muslims** like people fighting over a plate of food”
	Muslim_Counter	“This way people in general will come to love **Islam** and its message and will convert to this wonderful religion after having learnt its great principles and values”
A5	Extremist	“This is a **great** advantage for Muslims since during wars and fighting, their ranks will disunite and their assemblies will disintegrate”
	Muslim_Counter	“If you’re praying they stop killing innocent people, that’s **good**”
S7	Extremist	“For to try and defend oneself against criticism and blame in the New World **Order** today, from its Muslims and non-Muslims is indeed a waste of time”
	Muslim_Counter	“**Let** us put our dislike of Bush and his coterie of warmongering, torture-condoning neo-cons aside, and focus on what is really important-the future of our Iraqi brothers and sisters, who deserve nothing less than to live as free citizens, free from the evils of autocracy and the scourge of terrorism”
S2	Extremist	“If some **people** have in the past argued about the fact of the occupation, all the **people** of the Peninsula have now acknowledged it”
	Muslim_Counter	“It is their only battle, as they have no weapons except their own bodies and their own lives to resist the invasion of those who come with F-16s, tanks, and machine guns to kill their very own **children**”
E2	Extremist	“They **like** to spread mischief and corruption on earth and strive hard to accomplish this”
	Muslim_Counter	“I would **like** to recall here that the intolerant Catholics in Spain went very far against the teachings of Jesus himself, the prince of peace”
P1	Extremist	“They (Muslim **scholars**) say we have to obey our government, abide by its laws, serve in its military and security forces, and pay taxes”
	Muslim_Counter	“In fact, after September 11 and since, Muslim leaders and **scholars** have been voicing their condemnation of terrorism loud and clear”
S8	Extremist	“Raise your arms and **fight** to escape from this humiliation and shame!”
	Muslim_Counter	“I’d like to make it close to your mind why Muslims are in need of **fight** or combat”
S1	Extremist	“We know the truth about the leaderships of the first tier and their subjugation to our **enemies**”
	Muslim_Counter	“The second case why the ‘defensive’ acknowledged physical Jihad is when it brings about safety to the Muslim state and security its borders, especially when the state is being threatened by **enemies** who are plotting against it”

*Bold font corresponds to a word from a shared category.*

**TABLE 3:** Examples of shared conceptual categories between religious authored counter-extremist messages (Muslim_Counter) and British Official authored counter-extremist messages (BrOfficial_Counter).

**Category**	**Corpus**	**Example**
S6	Muslim_Counter	“Even if Spain and the UK were among the attackers, they **should** have fought against the soldiers who are in their countries, not random killing civilians, including children, who have no other fault than sitting in a train”
	BrOfficial_Counter	“Understand the causes of terror. Yes, we **should** try, but let there be no moral ambiguity about this: nothing could ever justify the events of 11 September, and it is to turn justice on its head to pretend it could”
X4	Muslim_Counter	“Somehow Al Qaeda has convinced Muslims that the only **way** to fight the West is through new means”
	BrOfficial_Counter	“You saw with Afghanistan or the 11th September attack, there’s no **way** Britain could have stood apart from that. I mean we could have taken a back seat, but we were still involved”
A13	Muslim_Counter	“The Prophet Muhammad said that anyone who killed **even** a bird unjustly would meet Allah on Judgment day”
	BrOfficial_Counter	“The **more** we reach out across the world of faith, the **more** common space the Abrahamic and non-Abrahamic faiths can inhabit, then the extremists and reactionaries within all faiths can be challenged”
A4	Muslim_Counter	“In **case** there is a violation to the security pledge by any non-Muslim citizen, then he is solely responsible for his personal violation, and no one except the Muslim ‘Extremist’ is allowed to question him for such violation”
	BrOfficial_Counter	“And here is why Iraq is important in this, because in the end their **case**, which is based on dividing people, the Arab world and the western world, the Muslim world and the Christian world and other religions, their **case** is that we are in Iraq to suppress Muslims, steal their oil, to spoil the country. Now we know, you know, that all those things are lies”
A7	Muslim_Counter	“Only God **can** guide individuals to Islam, not some disgusting fool named bin Laden”
	BrOfficial_Counter	“If we do take military action, we have to do everything we possibly **can** to minimise the civilian casualties”
A1	Muslim_Counter	“For this reason, Muslims **do** not encourage everybody to go about interpreting and explicating the Qur’an”
	BrOfficial_Counter	“The sceptics said it was pointless, we’d **make** matters worse, we’d **make** Milosevic stronger and look what happened, we won, the refugees went home, the policies of ethnic cleansing were reversed”
N5	Muslim_Counter	“Still, always the proviso is that fighting should be the last option, when **all** other avenues are closed”
	BrOfficial_Counter	“If international terrorism is defeated, we are **all** safer”
A14	Muslim_Counter	“The **only** difference between you and them is; they follow the Quran and the Sunnah, fearing Allah and not basing their judgments on their own opinions, while the others make their own conclusions according to their own desires”
	BrOfficial_Counter	“It turns upside-down our concepts of how we should act and when, and it crosses the frontiers of many nations. So **just** as it redefines our notions of security, so it must refine our notions of diplomacy”

*Bold font corresponds to a word from a shared category.*

**TABLE 4:** Examples of shared conceptual categories between British Official authored counter-extremist messages (BrOfficial_Counter) and Arab mainstream media messages (Mainstream).

**Category**	**Corpus**	**Example**
G1	BrOfficial_Counter	“The first priority of any **Government** is to ensure the security and safety of the nation and all members of the public”
	Mainstream	“The peace talks have also exacerbated tensions between Palestinian President Mahmoud Abbas’ West Bank **government** and the rival Islamic militant Hamas that rule the Gaza Strip and opposes negotiations with Israel”
M7	BrOfficial_Counter	“In the decades to come there will be many **international** negotiations, debates, occasionally, if only in a diplomatic sense, confrontations”
	Mainstream	“Kidnapping for ransom is common and a lucrative business in the Horn of Africa country and Somali fighters say they will stand up to the government until all **foreign** forces in the capital leave the country”
I2	BrOfficial_Counter	“There is now no contact permitted with western **agencies**, even those delivering food”
	Mainstream	“‘We observe the banks in the UAE, whether foreign or local banks, are applying more and more daily restrictions to the Iranian traders and businesses,’ said Morteza Masoumzadeh, the vice president of the Iranian Business Council (IBC) in Dubai and managing director of Jumbo Line, a shipping **agency**”
I3	BrOfficial_Counter	“It is right that we now also **work** more closely with allies in the region through a new ’Friends of Yemen’ group, we will help establish to pool effort, resource and expertise”
	Mainstream	“‘The idea that courts should have no **role** whatsoever in determining the criteria by which the executive branch can kill its own citizens is unacceptable in a democracy,’ the American Civil Liberties Union and Center for Constitutional Rights said. ‘In matters of life and death, no executive should have a blank check,’ they said”
A11	BrOfficial_Counter	“I think what is **important** is that we don’t just have a period of calm, but progressively, within that, we’re able to start to reopen the border crossings, get not just humanitarian aid in, but also get some of the business going in Gaza again”
	Mainstream	“The **main** alternative, according to officials, is to seek U.N. Security Council recognition of a Palestinian state in the West Bank, Gaza and east Jerusalem, the territories Israel captured in the 1967 Mideast war”
Y1	BrOfficial_Counter	“It is to prevent Iran acquiring **nuclear** weapons capability; but it is more than that, it is to put a stop to the Iranian regime’s policy of de-stabilisation and support of terrorism”
	Mainstream	“A new U.N. nuclear agency report shows that Tehran has now amassed nearly twice as much enriched uranium as the West wants removed from Iran. That finding is likely to increase Western opposition to a **nuclear** deal that Iran says would build trust about its atomic activities”
Y2	BrOfficial_Counter	“Whereas once, influence was carried by word of mouth and through books and newspapers, today the **internet** and 24 h media allow access to a global audience with examples of course of young people being radicalised solely by contact with the internet”
	Mainstream	“The Zionist regime’s ambassador to the UN Gabriela Shalev sent a letter to Secretary General Ban Ki-moon asking that the international community intervene to prevent the ship approaching Gaza, the **website** of the Israeli regime paper Haaretz daily reported”
S5	BrOfficial_Counter	“The world **community** must show as much its capacity for compassion as for force. The critics will say: but how can the world be a **community**? Nations act in their own self-interest. Of course they do. But what is the lesson of the financial markets, climate change, international terrorism, nuclear proliferation or world trade?”
	Mainstream	“After democratic elections last year, the government formed by **Hamas** was paralysed by a punishing Western aid freeze and the withholding by Israel of Palestinian tax revenue”
M5	BrOfficial_Counter	“Likewise, we must see what more scope there is to contract **helicopters** commercially to do some of the routine tasks, and free up helicopters for the frontline”
	Mainstream	“China is a strong ally of Pakistan and Islamabad draws heavily on Beijing for its defense and infrastructure needs. Pakistan’s air force has a fleet of Chinese **aircraft**, including F-7PGs and A-5s, but also U.S.-built F-16s and French Mirages”
K1	BrOfficial_Counter	“If Europe and America are together, the others will work with us. If we split, the rest will **play** around, **play** us off and nothing but mischief will be the result of it”
	Mainstream	“The Israeli air force **played** a key role in a fierce three-week offensive in Gaza early last year, which began with airstrikes that killed hundreds of Hamas fighters”
X6	BrOfficial_Counter	“in conflict **resolution**; encouraging investment; and access to our markets so that we practise the free trade we are so fond of preaching”
	Mainstream	“Azizi added that the demolition is also motivated by the government plan to take advantage of the priceless land on which the palace was located. ‘Because of the corruption that pervades its institutions, the Revolutionary Guard is not only dominating political **decision** making but also the economy”’

*Bold font corresponds to a word from a shared category.*

Examples were then subjected to a positioning analysis. We used Bamberg’s (1997, p. 341) perspective on positioning, which views this as “the speaker’s active engagement in the construction process of narratives.” This construction process consists of three levels (Bamberg, 1997, p. 337):

**Level 1:** This level entails looking at linguistic devices which indicate how characters are being positioned relative to one another within a series of reported events. Specifically, this includes an examination of agency, i.e., who is marked as being in control of the action? Who is acted on by external forces or rewarded by their personal qualities?

**Level 2:** This level looks at how the narrator positions themselves relative to their audience by way of a linguistic analysis of attempts to instruct the audience “in the face of adversary conditions,” or otherwise make excuses or attribute blame for their actions to others.

**Level 3:** This level looks at the narrator’s construction of their own identity (identity claims), specifically, how they answer (indirectly) the question of who they are. This element of the analysis moves beyond the language used to what the narrator holds to be true beyond the local situation.

Each of these levels were employed on the examples listed in Tables 1–4. Within tables, similarities in positioning were taken to indicate shared narratives between the two message types, while differences in positioning were taken to indicate individual narratives, or narratives shared with another message type. The latter was ascertained by looking at similarities in positioning observed across Tables 1–4.

## Results

This section provides a summary of the results of the keyness comparison and semantic concordance analyses. Table 5 presents a numerical breakdown of the conceptual categories held or shared between different message types.

**TABLE 5:** Showing numerical breakdown of shared conceptual categories between message types.

**Corpus/Corpora**	**No. shared categories**	**% of categories**
Extremist/Muslim_Counter/Mainstream/BrOfficial_Counter	27	25.96%
Extremist/Muslim_Counter	8	7.69%
Extremist/Mainstream	6	5.77%
Extremist/BrOfficial_ Counter	3	2.88%
Muslim_Counter/Mainstream	3	2.88%
Muslim_Counter/BrOfficial_Counter	8	7.69%
Mainstream/BrOfficial_Counter	11	10.58%
Extremist/Muslim_Counter/mainstream	0	0.00%
Extremist/Mainstream/BrOfficial_Counter	2	1.93%
Muslim_Counter/Mainstream/BrOfficial_ Counter	1	0.96%
Extremist/Muslim_Counter/BrOfficial_ Counter	3	2.88%
Extremist	6	5.77%
Muslim_Counter	1	0.96%
Mainstream	6	5.77%
BrOfficial_Counter	2	1.93%
Total	87 (of 104)	83.65%

Table 6 presents a breakdown of the categories shared by the most frequently occurring message groupings: all four message types, British Official counter messages and Arab mainstream media messages, extremist messages and religious authored counter messages, and British official counter messages and religious authored counter messages.

**TABLE 6:** Listing shared conceptual categories between selected groups of message types.

**Extremist, Muslim_Counter, mainstream, and BrOfficial_Counter**		
L3. Plants	N6. Frequency	X7. Wanting, planning, choosing
B4. Cleaning and personal care	S3. Relationship	A15. Safety/danger
K2. Music	X5. Attention	K4. Drama and the theater
K5. Sports and games	W4. Weather	W2. Light
E6. Worry, concern, confidence	X8. Trying	O4. Physical attributes
A8. Seem	F4. Farming and horticulture	H5. Furniture and household
N4. Linear order	I4. Industry	I1. Money generally
H4. Residence	M8. Remaining/stationary	
E1. Emotional actions and states	A10. Open/closed, hidden/hiding	
H3. Areas around/near buildings	F2. Drinks	

**BrOfficial_Counter and mainstream**	**Muslim_Counter and extremist**	**Muslim_Counter and BrOfficial_Counter**

G1. Government and politics	S9. Religion	S6. Obligation and necessity
M7. Places	A5. Evaluation	X4. Mental object (means, method)
I2. Business	S7. Power relationship	A13. Degree
I3. Work and employment	S2. People	A4. Classification
A11. Importance	E2. Liking	A7. Definite
Y1. Science and technology	P1. Education in general	A1. General actions
Y2. IT and computing	S8. Helping/hindering	N5. Quantities
S5. Groups and affiliation	S1. Social actions, states and processes	A14. Exclusivisers/particularisers
M5. Movement and transportation: air		
K1. Entertainment generally		
X6. Deciding		

Tables 1–4 present examples of shared categories from the corpus groupings featured in Table 6. Table 1 provides concordance examples of the categories shared between all four message types.

Table 2 provides concordance examples of the categories shared between the Salafi Jihadist and related messages and the religious authored counter messages.

Table 3 provides concordance examples of the categories shared between the religious authored counter messages and British Official authored counter messages.

Table 4 provides concordance examples of the categories shared between the British Official authored counter messages and the Arab mainstream media messages.

## Discussion

This section discusses the results of the keyness comparison and semantic concordance procedures presented in Tables 1–6.

### Extent and Nature of Overlap of Conceptual Categories

Of the 104 categories included in the analysis, 40 categories (38.46% of 104 categories) received negative BIC values, with 27 categories (25.96%) returning a BIC ≤ –10 and all corpus frequencies > 5, indicating no discernible difference between the usage of these categories across the message types. The remaining 64 categories returned positive BIC values, of which 60 returned BIC values above 10 and 4 returned values between 1.69 and 8.84. Table 5 presents a breakdown of the categories above the specified threshold, i.e., LL value ≥ 15.13 and a BIC value ≥ 10, or BIC value ≤ –10 and all corpus frequencies > 5.

The results presented in Table 5 suggest that around a quarter of the conceptual categories are shared by all message forms. This is followed by British Official counter messages and Arab mainstream media messages, which interestingly demonstrate a greater degree of overlap than Religious authored counter messages and Arab mainstream media messages (10.58%, compared with 2.88%). The next highest number of shared categories are found between the extremist and Religious authored counter messages, and British Official and Religious authored counter messages, both of which share the same number of categories at 7.69% each. Therefore, Religious authored counter extremist messages and extremist messages are as close in conceptual terms as both forms of counter message are to one another. Importantly, extremist material does not stand out in these comparisons.

While the results provide an element of empirical support for Al Raffie’s (2012) argument that mainstream narratives adopt the same master narrative as extremist messages, in that both Religious authored and Arab based mainstream media messages demonstrate some overlap with extremist material, this overlap is not as extensive as the overlap between all four message forms and no more extensive than the overlap between Religious authored counter messages and British Official counter messages, or British Official counter messages and Arab mainstream media messages (indeed, less so than the latter).

Given these observations, Religious authored counter messages could also be argued to be simultaneously borrowing from a Western master narrative, or vice versa, as indeed, could Arab mainstream media. The observation that different groups of messages overlap to differing degrees suggests a complex blend of narratives. Looking at the results presented in Table 6, one can begin to unpick the complexities between the groups of messages.

The categories shared by all four message forms are varied in nature and include concepts related to emotion (Worry, Concern, Confidence; Emotional Actions and States), thought processes (Attention; Trying; Wanting, Planning, Choosing), residence (Residence; Areas Around/Near Buildings; Remaining/Stationary; Furniture and Household), and a series of categories that one might not expect, such as Plants, Weather, Light, Cleaning and Personal Care, Sports, Music, and Drama. Such categories may be indicative of shared metaphorical language use. There are also categories which point to narrative structure (Linear Order) and interpretation or evaluation (Seem; Open/Closed, Hidden/Hiding, Finding/Showing; Physical Attributes).

The categories shared by the British Official counter messages and Arab mainstream media messages appear to be in large part driven by business, industry and the economy. These categories would tend to suggest a capitalist master narrative, which may suggest that Arab mainstream media is borrowing from this narrative. Similarities between Religious authored counter messages and extremist messages are drawn on social grounds, with most of the categories falling under the domain of “Social Actions, States and Processes,” according to the automated semantic categorization system used. Categories overused by both the Religious authored counter messages and British Official counter messages are more what one might describe as surface deep, referring mainly to categories that define the scale or bounds of something, or otherwise belong to the domain of “General and Abstract Terms” within the USAS classification scheme. These categories refer to actions.

To understand whether or not these initial observations mean that one message form is borrowing from the master narrative of another, one needs to look deeper into the data and explore how authors position themselves and others in relation to the conceptual categories and beyond. In other words, one needs to apply the three levels of narrative analysis outlined in the section “Semantic Concordance Analysis” to the results presented in Tables 1–4.

### Positioning Analysis of Overlapping Categories

In Table 1, the examples of categories B4.Cleaning and Personal Care and L3.Plants provide evidence of shared metaphor use between the message types. The extremist and Religious authored counter messages share metaphors of cleansing, with Religious authored counter messages speaking of the need to clean the soul, while extremist messages liken hypocrisy to dirt that one struggles to “wipe off.” Meanwhile, Arab based mainstream media and British Official counter messages make frequent use of brushing or sweeping metaphors to reference issues that cannot be ignored and, by implication, must be dealt with. Interestingly, all message forms make use of the metaphor of the tree. However, this is utilized for different purposes.

In extremist messages, the tree metaphor is often used to describe Muslims and is embedded in tree symbolism present in Islam, which Reat (1975, p. 2) describes as “a universal symbol of order in the midst of chaos.” In this case, as with British Official counter messages referenced below, the extremist message author here positions their audience as a disparate one, using the tree metaphor as a means of expressing a desire to restore order. In Religious authored counter messages, Arab mainstream media messages and British Official counter messages, the tree (or plant) is used as a means of representing terrorism or the aggressor, who has roots and branches, grows and must be uprooted or “pulled out.” The mainstream example mixes this metaphor with one of disease (see use of the word “microbe”). In mainstream messages (and, indeed, in other message forms), this category can also be used to literally refer to trees. In mainstream messages, this particularly applies to olive trees, which are a source of contention and conflict between Israelis and Palestinians.

Whilst Religious authored counter messages and British Official counter messages may share similar metaphor use, the positioning in example Muslim_Counter L3 reveals that, while terrorism is perceived as a mutual issue, for religious counter message authors, governments can also be seen as part of the problem. By placing “civilized world leaders” in quotation marks, the author simultaneously distances themselves from such individuals and questions their integrity, underlining this with use of the pronoun “they” (a further distancing strategy) before referring to leaders not wanting to hear about the “true” causes of terrorism, thus implying that world leaders are dismissive and refuse to acknowledge their role in the problem.

With regard to narrative structure, which is indicated by use of the category N4.Linear Order, one can observe that both the Religious authored counter messages and extremist messages most commonly use the word “then.” However, for extremist message authors, this tends to be used for the purpose of listing events in chronological order, which emphasizes the out-group’s continued interference (in this case, collaboration between the United States and Iran in relation to the Taliban). In Religious authored counter messages, authors tend to use “then” as a means of reasoning with their audience, i.e., if X then Y. Arab mainstream media and British Official counter messages most frequently use the words “last” and “first,” respectively. In British Official counter messages, “first” is generally used to mark an order of prioritization, while in mainstream media, “last” is used either as a marker of finality (as illustrated in Table 1), or to refer to past events that have relevance to the present (e.g., “last month”).

There are similarities demonstrated between the Religious authored counter messages, Arab mainstream messages and extremist messages with respect to categories X7. Wanting, Planning and Choosing and H4.Residence. In category X7, all three of these message types refer to the desire for Muslims to lead a quality life, while in category H4, Muslims are positioned as the recipients of external aggression. In category E1.Emotional Actions and States, however, Religious authored counter messages refer to a cultural master narrative of tolerance and compassion that can be traced back through history, while extremist message authors tend to use this category to highlight the positive morale felt by their own in-group of fighters, linking this morale to the morale felt by those fighting against oppression, as described in the Quran. In British Official examples for categories H4, E1 and X7, there is a sense in which the authors are speaking to a disparate audience. The “our” referred to in British Official counter message example X7 consists of a range of different communities rather than a single unified one, which requires effort to maintain (as indicated by, “we have to work at”).

In Table 2, extremist and counter-extremist authors position themselves in a similar way with regard to the state of Israel (this is one of the “enemies” referred to in example Muslim_Counter S1 and is the “They” referred to in example Extremist E2) and political interference in Iraq (see Muslim_Counter S7), with both referring to underhand dealings or corruption on the part of those in power, see “plotting against it” and “the truth” in Extremist S1 and Muslim_Counter S1. Both sets of authors position themselves as members of the Muslim community. However, the authors are not addressing themselves to the same audience.

The Extremist S7 example positions certain members of the Muslim community (scholars, leaders of particular Arab nations) within what it refers to as “the New World Order” and sets “Muslim scholars” firmly in the out-group with “They say we have to” (Extremist P1). Here, the “we” refers to the general Muslim public, of which particular Muslim scholars are not seen to be a part. Meanwhile, counter-extremist authors identify themselves as Muslim scholars *and* as being a member of their Muslim community and place terrorists (those who attack non-combatants) on a par with autocratic leaders (see example Muslim_Counter S7).

The examples presented in Table 3 largely corroborate the initial interpretation of similarities between the British Official (BrOfficial_Counter) and Religious authored counter messages (Muslim_Counter), in that both define the boundaries of physical action, boundaries that are not too dissimilar from one another. Both argue for having no choice but to act in the face of a perceived aggressor. See, for example, BrOfficial_Counter X4 in Table 3 and Muslim_Counter S2 in Table 2. A number of the BrOfficial_Counter examples speak to a master narrative of securitization (for example, BrOfficial_Counter A14), i.e., framing terrorism as an issue of security and counter-terrorism as a means of protecting the “safety” or “security” of one’s in-group and the borders of that in-group, which has been said to define European political responses to terrorism (Tsoukala, 2006).

Nevertheless, the Religious authored counter-extremist messages also speak to the concepts of security and safety in defining the boundaries of action, see, for example, Muslim_Counter S1 in Table 2 and Muslim_Counter A4 in Table 3. However, for religious counter message authors, these boundaries are defined for them by the word of Allah and Islam’s religious scripture. From this perspective, only these sources should dictate action and not external forces or individual opinions (see, for example, Muslim_Counter A14, A1 and A7), and therefore one cannot take matters into one’s own hands (see Muslim_Counter A4).

One can again observe, via the positioning present in examples, that British Official counter-extremist messages and Religious authored counter-extremist messages do not identify themselves as members of the same in-group or address the same audience. Example BrOfficial_Counter A4 is a good example of this positioning. When the author states “their case is that,” they refer to extremists, setting these individuals firmly in the out-group category. However, the author is addressing the Muslim community at large and goes on to state “we know” (i.e., Western nations), “you know” (i.e., “Muslim communities”). While this statement suggests solidarity, it still separates Muslims from the author’s in-group. In other examples (such as BrOfficial_Counter A14), British Official counter authors address their messages to the entire British public, referring to “our concepts” and “our notions.” However, this assumes that all members of the British public share these concepts and notions, which are based on a system of Western values.

In a similar way, Religious authored counter messages also set extremists as the outgroup, such as in example Muslim_Counter S6 (“they should have fought”) and Muslim_Counter A7 (in which Bin Laden is labeled a “disgusting fool”). However, the West, and nations within this sphere, are also described in a manner that is outside the authors’ in-group and something that requires resistance, see for example, Muslim_Counter S6 and Muslim_Counter X4. Note that within the statement “has convinced Muslims [in-group] that the *only* way to fight the West [out-group],” the use of the adjective “only” infers that there are other ways to fight or resist the West.

The examples presented in Table 4 show that there is a degree of mainstream English language Arab media borrowing from a capitalist master narrative, with references to the economy (Mainstream X6), defense and infrastructure (Mainstream M5), tax revenue (Mainstream S5), and forms of business and trade (Mainstream I2 and Mainstream M7). Further, the mainstream messages report on stories of concern to the West, such as the Iranian nuclear enrichment program (see example Mainstream Y1).

However, there is another key point of cross over between the message forms, in that British Official counter-extremist messages contain narratives of resistance, while Mainstream Arab English language media reports narratives of resistance, whether in a direct or indirect manner. Examples Mainstream X6 and Mainstream M7 give voice to those challenging government control. Voices are also given to those resisting trade embargoes (Mainstream I2) or capital punishment (Mainstream I3). The mainstream messages further report narratives of opposition between groups, including in examples Mainstream G1 and Y1.

Mainstream message positioning also reveals its similarities to both extremist and Religious authored counter messages with regard to resistance to Israel and positioning the West as an out-group. In Mainstream S5, the article’s author points out that the election of Hamas was “democratic” and describes Western actions in response as “punishing.” Israel is referred as a “Zionist regime” in Mainstream Y2. In example Mainstream A11, the author states, “the main alternative, *according to officials*” (in relation to peace negotiations between Israel and Palestine), thereby distancing the author from this view. The way in which authors refer to out-group actors and frame the actions of out-group members is demonstrative of a more indirect form of resistance.

### Practical and Theoretical Implications

This paper set out to empirically test the hypothesis that non-extremist narratives overlap with a Salafi Jihadist master narrative (and those of similar groups and individuals), specifically, the argument that “Mainstream Islamic narratives indirectly support the master narratives of Salafi Jihadists because in some instances there exists considerable overlap between the two” (Al Raffie, 2012, p. 22). The results of the quantitative comparative analysis provided some support for this hypothesis, revealing that Salafi Jihadist and related material only significantly differed from all forms of non-extremist material considered on around 6% of conceptual categories that were examined. However, this analysis included British Official counter messages and showed that 25% of categories were shared by all message forms.

Nevertheless, the analysis demonstrated conceptual overlap between extremist messages and both Religious authored counter messages and Arab mainstream media messages on selected sets of categories. Though the extent of overlap between these particular message forms was not demonstrably different from the extent of overlap between Arab mainstream media messages and British Official counter messages, or Religious authored counter messages and British Official counter messages.

While the subsequent qualitative positioning analysis did further corroborate elements of similarity between the narratives used in extremist and non-extremist material, it further revealed a series of nuanced differences that were obscured by the quantitative comparison. These nuanced differences pointed to multiple layers of positioning, which are said to characterize counter narratives (Bamberg and Andrews, 2004, p. x). If one considers all message types included in the present analysis as forms of counter, or resistance narrative, then one begins to better understand the similarities between these forms of material. Message forms may practice their resistance in a direct and overt manner, or more indirectly (as is the case with mainstream media reporting, which does so via giving voice to resistance, reporting on resistance, or via editorial labeling and story framing).

Sometimes the master narratives that groups are opposing are the same. Religious authored counter messages and extremist messages, for example, both oppose a narrative of Western dominance, while Religious authored counter messages and British Official counter messages both oppose an extremist narrative that actively calls for violence against civilians/non-combatants. However, the message forms also demonstrate their own narratives of resistance, identifying with their own in-groups, addressing their own audiences and defining their own out-groups. The final section of this paper will expand on why each of the message forms can be seen as a form of resistance narrative, and what implications this finding has for counter-extremism policy.

Andrews (2004) defines counter-narratives as “the stories which people tell and live which offer resistance, either implicitly or explicitly, to dominant cultural narratives.” In this respect, extremist messages are themselves a form of counter-narrative, offering resistance to a dominant Western cultural narrative and anyone identifying as a Muslim who adopts any aspect of this master narrative. Indeed, HM Government’s (2013, p. 1) Prevent strategy defines extremism as a form of opposition, i.e., as “vocal or active opposition to fundamental British values, including democracy, the rule of law, individual liberty and mutual respect and tolerance of different faiths and beliefs.”

As the analysis in this paper has demonstrated, both Religious authored counter messages and Arab mainstream media messages can also be observed to resist elements of a dominant Western master narrative, just as extremist message authors can be found to align with elements of this narrative, albeit with an alternative framing. For example, extremist message authors also refer to a desire for freedom and liberty, but their perspective on what this entails and the manner through which it is achieved differs from British Official authors. As Andrews (2004) argues, “counter-narratives exist in relation to master narratives, but they are not necessarily dichotomous entities.” A group may borrow elements of a particular master narrative, while resisting others. Mainstream messages may borrow elements from a capitalist master narrative, but reject other elements of capitalist societies, while Religious authored counter messages may, like extremist messages, borrow from a cultural master narrative of fighting oppression, but reject elements that argue for the fighting of non-combatants.

Further ways in which the message forms can be seen as forms of resistance narrative emerge from specific elements of their linguistic performance. Sandberg and Andersen (2019, p. 445) interviewed a set of participants to investigate counter-narratives to those of jihadist extremist organizations, referring to the narratives they observed as “narrative resistance to master narratives that describe Islam as a religion of war and terrorism.” Among the resistance narratives the authors observed were “criticizing extremist jihadist organizations for false interpretations of Islam and using derogatory terms to describe them.” Note that these observations bear similarities to extremist message authors’ descriptions of what they refer to as “sham” or “bogus” scholars, whom they perceive as incorrectly interpreting their religion.

If one views extremist messages as a form of resistance narrative, what does this mean in practical terms for counter-extremism policy? Literature on resistance narratives offers us potential insights. In relation to resistance narratives, Andrews (2004, p. 1) states that:

“When, for whatever reason, our own experiences do not match the master narratives with which we are familiar, or we come to question the foundations of these dominant tales, we are confronted with a challenge. How can we make sense of ourselves, and our lives, if the shape of our life story looks deviant compared to the regular lines of the dominant stories? The challenge then becomes one of finding meaning outside of the employments which are ordinarily available. We become aware of new possibilities.”

Extrapolating from this statement, some individuals may find meaning in extremism (whether framed in religious terms or otherwise), which is turned to as a means of resisting a dominant narrative into which they do not fit. Framed in this way, countering extremism becomes a question of individual identity. How do individuals make sense of themselves and how do they see themselves in relation to dominant cultural narratives? Given an understanding of this, how can we assist the individual in finding meaning and what positive new possibilities might be offered to the individual as a result?

In practical terms, this could involve investment in, or capitalizing on, grass-roots projects and initiatives that seek to understand the layered nature of individuals’ identities, and to guide individuals toward roles and outlets that allow them to explore and exercise these identities. At a national level, the observations made here problematize top-down attempts to define a singular, unified national identity and associated values within counter-extremism policy, in that such efforts impose a dominant perspective that could be said to generate resistance from those who do not perceive themselves to fit the defined frame; individuals one might wish to engage with. Instead, a starting point might be to draw on the aforementioned projects and initiatives to co-create a bottom-up definition of national identities (plural) and values, which overtly recognizes and acknowledges the complexities, oppositions and tensions at play.

This paper concludes with a caveat. Whilst this piece has provided insights into the overlaps between extreme and non-extreme message content, it is worth highlighting that there are limitations to the methodology and analysis techniques used. The analysis entailed a detailed reading of concordance examples and the reporting of illustrative examples of patterns and trends observed within these examples. Nevertheless, one might argue that the insights provided are surface-deep in nature. Future work should look to explore similarities in content further, for example, by taking sets of texts from each of the message types which contain a high number of the overlapping concepts identified here and exploring whether such texts employ similar arguments and rhetorical strategies. One possibility would be to explore whether non-extreme messages with conceptual similarity to extremist messages employ the types of strategies previously identified in studies of extremist messages (see Prentice et al., 2011). Such an analysis would strengthen the connection between conceptual and rhetorical similarity.

## Data Availability Statement

The datasets presented in this article are not readily available because of the nature of the material collected and analyzed. Permission to use the material can be obtained via a data sharing agreement with Lancaster University. Requests to access the datasets should be directed to PT at p.j.taylor@lancaster.ac.uk.

## Ethics Statement

The studies involving human participants were reviewed and approved by the Faculty of Science and Technology (FST) Ethics Committee, Lancaster University, United Kingdom. Written informed consent for participation was not required for this study in accordance with the national legislation and the institutional requirements.

## Author Contributions

SP was responsible for the first draft of the manuscript, collection of the mainstream and extremist datasets, partial collection of the counter-extremist dataset, and analyses. PT has provided subsequent additions and amendments and co-created (with SP) the idea for this study and also supplied some of the analysis material. Both authors contributed to the article and approved the submitted version.

## Conflict of Interest

PT holds a position as Chief Scientific Advisor on Policing at the NPCC. SP is a Director of W&P Academic Consultancy Limited. Both authors have previously received funding from HMG, United Kingdom.

## Publisher’s Note

All claims expressed in this article are solely those of the authors and do not necessarily represent those of their affiliated organizations, or those of the publisher, the editors and the reviewers. Any product that may be evaluated in this article, or claim that may be made by its manufacturer, is not guaranteed or endorsed by the publisher.
